# The Screening Visual Complaints questionnaire-acquired brain injury: Development and evaluation of psychometric properties in a community sample

**DOI:** 10.1371/journal.pone.0314999

**Published:** 2024-12-31

**Authors:** Vera Linde Dol, Anselm B. M. Fuermaier, Eline M. E. Will, Arlette J. van Sorge, Joost Heutink

**Affiliations:** 1 Faculty of Behavioural and Social Sciences, Department of Clinical and Developmental Neuropsychology, University of Groningen, Groningen, The Netherlands; 2 Royal Dutch Visio, Center of Expertise for Blind and Partially Sighted People, Huizen, The Netherlands; Universiti Sains Malaysia, MALAYSIA

## Abstract

**Purpose:**

Visual complaints are a common consequence of acquired brain injury (ABI). Yet, they may be overlooked in clinical practice. The present study aims to develop a screening instrument for assessing visual complaints in individuals with ABI and evaluate its psychometrics properties in a community sample.

**Methods:**

We constructed the Dutch Screening Visual Complaints questionnaire-acquired brain injury (SVCq-abi), a self-report 23-item questionnaire. A Dutch community sample of 1159 participants (Mean [SD] age, 60 [16] years) completed the SVCq-abi and other questionnaires on visual disturbances, executive functioning, mental health, and autistic traits. Confirmatory factor analyses were performed for 5 models (1-factor, 3-factor, 5-factor, second-order, and bifactor) on a random split of half of the sample, and cross-validated on the other half. In addition, we evaluated floor and ceiling effects, scale reliability, test-retest reliability, as well as convergent and divergent validity.

**Results:**

A 5-factor structure of the SVCq-abi was adopted which showed an excellent model of fit, with all item loadings exceeding 0.5. The SVCq-abi subscales demonstrated various floor effects, acceptable scale reliability, moderate to good test-retest reliability (ICC = 0.65 to 0.73), along with sufficient convergent (r^2^ = 9% to 32%) and divergent validity (r^2^ = 2% to 13%).

**Conclusions:**

The SVCq-abi shows fundamental psychometric properties and the factor analysis provides support for a 5-factor structure. Further validation of the SVCq-abi in individuals with ABI is essential.

## Introduction

Acquired brain injury (ABI) encompasses non-congenital brain injuries and can be the result of traumatic brain injury, stroke, tumor, infection and anoxia [1,2]. Problems with vision are a common consequence of ABI and have been documented in approximately 50 to 75% of individuals with ABI [3–9]. Often reported visual problems are reading disturbances, visual field defects, perceptual disorders, diplopia, depth impairment, and photophobia. These problems can significantly impact quality of life and independence, and may hamper rehabilitation processes [8,10–13]. Despite their impact, the visual consequences of ABI are often underestimated and overlooked in clinical practice [3].

A potential reason for this underestimation could be that many patients with ABI may not report visual problems spontaneously unless specifically questioned [3,14–16]. Moreover, screening for visual problems is not embedded in standard care in Dutch rehabilitation centers. Rehabilitation clinicians refer patients to an ophthalmologist or specialized visual rehabilitation center when they suspect visual dysfunctions are present, yet, visual problems may not always be visible to an observer [17]. These findings underline the need for vision screening to improve timely detection of visual problems in order to adapt further care accordingly, and refer to a specialist center when necessary.

While screening tools using tests of visual function and perception have been developed [18–20], these tools often require training and can take over 20 minutes to administer, making them less suitable for routine screening. Moreover, it is not clear if objective measures capture the full extent and range of vision-related problems individuals with ABI may experience [16,21,22]. Questionnaires, designed to capture the individual’s complaints and needs, may offer a practical alternative. However, current questionnaires may fall short in measuring some important visual domains (e.g., Cerebral Vision Screening questionnaire; CVSQ [23,24]) or focus on assessing the impact of visual impairments rather than assessing complaints at a functional level (e.g., Brain Injury associated Visual Impairment—Impact Questionnaire; BIVI-IQ-15 [25]).

Therefore, the aim of this study was to develop and validate a questionnaire to screen for visual complaints in individuals with ABI. For this purpose, we constructed the Screening Visual Complaints questionnaire-acquired brain injury (SVCq-abi), a self-report 23-item questionnaire. The SVCq-abi is an adaptation of the Screening Visual Complaints questionnaire (SVCq), a screening instrument that measures visual complaints in individuals with neurodegenerative diseases [26–28]. Psychometric properties were evaluated in a large Dutch community sample, including the factor structure, floor ceiling effects, scale reliability, test-retest reliability, as well as convergent and divergent validity.

## Methods

### Questionnaire development

#### Item bank generation

We constructed an item bank with items describing complaints potentially relevant to individuals with ABI experiencing visual problems. We reviewed literature and relevant validated measures, including the SVCq [26–28], CVSQ [23,24], BIVI-IQ-15 [25], Vision Interview (VI; [3]), and the Brain Injury Vision Symptom questionnaires (BIVSS; [29]). We focused on items that measure complaints on a functional and activity level, ensuring accessibility for both inpatient and outpatient settings. In addition, a multidisciplinary focus group with professionals from visual rehabilitation and ophthalmology (neuropsychologist, occupational therapists, and ophthalmologist) formulated new items based on clinical experience. All items were categorized into constructs summarizing the focus of the items (e.g., both ‘SVCq item 8’ and ‘CVSQ item 8’, which assess color vision, were categorized under the same construct), resulting in a bank of 88 items categorized into 28 constructs. Each construct contained between 1 and 14 items.

#### Ranking exercise and version 1 development

The focus group ranked all constructs from most important to least important for screening for visual problems in individuals with ABI. Subsequently, for each construct a key item was chosen. Given that the key items were predominantly derived from the SVCq, we opted for the SVCq as a base for the new instrument. Some SVCq items (n = 12) were modified to better fit the ABI population. Furthermore, based on the results of the ranking, some SVCq items were excluded (n = 2) and new items were added (n = 3). This process resulted in Version 1 of our questionnaire, comprising 21 items in total.

#### Version 1 pretest

Three rehabilitation professionals reviewed Version 1 for clarity and completeness and provided feedback. In addition, a pretest of Version 1 was done to identify difficulties encountered when completing the questionnaires and to verify whether they interpreted the questions as they were conceptualized. Six participants, including 3 participants without ABI and 3 individuals with ABI that followed a rehabilitation trajectory at a visual rehabilitation center, completed Version 1 by means of a cognitive interview [30,31]. All participants were over 18 years of age and provided written informed consent. In sum, the cognitive interview implied that (step 1) participants were asked to think aloud while completing the questionnaire, their behavior and thoughts were observed (skipping questions, corrections of chosen response category, hesitation). Subsequently (step 2), participants were asked to provide background on behavior observed by the researcher, and asked for opinions and experiences with regard to the questionnaire. At this stage, participants were also specifically asked for their opinion on the differences between the original SVCq and the adapted version of the instrument for individuals with ABI. Finally, in case they felt certain complaints or aspects of visual problems were missing, the participants were given the opportunity to suggest new items.

#### Version 2 construction

Based on the pretest and provided feedback, we made some modifications (including rephrasing questions, splitting a question into two, and changing the order of the questions) and determined Version 2 of the questionnaire, i.e. the SVCq-abi. The SVCq-abi includes 23 items, with 21 items identical or similar to those in the SVCq.

### Study population

Participants comprised a convenience sample of the Dutch population, stratified by age and gender. We aimed to include a sample that roughly matched the age and gender distribution of an ABI population in rehabilitation settings, to enhance generalizability of results to the ABI population. All participants were older than 18 and had Dutch as their primary language.

### Data collection

Participants were recruited between May 26, 2023 and June 29, 2023 via PanelInzicht. PanelInzicht is a Dutch online research panel focused on online quantitative data collection. We required a minimum sample size of 1,000 participants, as larger sample sizes enhance the accuracy of validity testing and facilitate the splitting of the sample for cross-validation of the factor models. Participants were invited to complete the SVCq-abi online, along with the CVSQ [23,24], Autism-spectrum Quotient-short (AQ-short) [32], the Depression Anxiety Stress scale-21 (DASS-21) [33], and the Behavioural Rating Inventory of Executive Function-Adult (BRIEF-A) [34]. Upon completion, participants received a small financial compensation. The estimated time for completing all questionnaires was around 15 minutes. Participant characteristics, including age, gender, education level, presence of severe ophthalmic conditions and presence of neurological conditions, were collected based on self-report. To evaluate test-retest reliability, a random subset of the sample was invited to complete the SVCq-abi and BRIEF-A again after a minimum of one week. Time to complete both questionnaires was estimated around 5 minutes.

The Ethical Committee Psychology of the University of Groningen (UG) approved the study protocol on June 22, 2022 (#PSY-2122-S-0162). All participants provided written informed consent.

### Materials

#### SVCq-abi

The SVCq-abi is a 23-item self-report questionnaire that aims to measure visual complaints in individuals with ABI (see S1 Appendix for the original Dutch questionnaire that was used in the present study; see S2 Appendix for an English translation). The SVCq-abi is an adaptation of the SVCq; a measure originally developed to measure visual complaints in individuals with neurodegenerative diseases [26–28]. A 3-factor structure was evidenced for the SVCq in a community sample, while further validation demonstrated a better fit of a 5-factor structure in individuals with multiple sclerosis and Parkinson’s disease. The 5-factor structure comprised the following subscales: *function related*, *luminance related*, *task related*, *altered visual perception*, and *ocular discomfort*.

The SVCq-abi starts with a semi-structured inventory question asking if visual complaints are present (item 1), employing a 3-point Likert scale (‘No/rarely (0)’, ‘sometimes (1)’, ‘often/always (2)’). If complaints are present (‘sometimes’ or ‘often/always’), respondents are asked to specify these complaints. The main body of the SVCq consists of 21 structured items (item 2 to item 22), each describing a visual complaint, and utilizes the same 3-point Likert scale. The final question, item 23, gauges the degree of limitations experienced in daily life resulting from the reported visual complaints, with response options ranging from 0 ‘no limitations’ to 10 ‘very severe limitations’. Scores for the main body of the SVCq-abi can be calculated by summing up the 21 items, with higher scores indicating higher frequency or severity of complaints.

#### CVSQ

We used the translated Cerebral Vision Screening questionnaire, which was later modified by Dittrich [23,24,35]. Although the validity of the version of Dittrich is not known yet, the original CVSQ by Kerkhoff et al. showed good psychometric qualities. The CVSQ assesses visual complaints in individuals following stroke. The questionnaire comprises eight questions related to the presence of visual disabilities, with scoring ‘yes (1)’/‘no (0)’ / ’not applicable (NA)’ (Kerkhoff et al.-part), and 12 activity-related questions on a 5-point Likert scale with answer options ‘no problem (0)’ to ‘mostly a problem (4)’ (Dittrich-part). For each part, a score was calculated by summing up the items, with higher scores indicating higher visual disability. A composite score was formed by summing up all items.

#### AQ-short

The AQ-short is an abridged version of the 50-item Autism spectrum Quotient, a self-report questionnaire that assesses autistic trains in individuals with normal intelligence [32]. The AQ-short consists of 28 items comprising two higher order factors assessing social behavioral difficulties (*social behavior*) and fascination for numbers and patterns (*numbers/patterns*). Participants are asked to respond to statements on a 4-point Likert scale, with response categories ‘definitely agree (1)’, ‘slightly agree (2)’, ‘slightly disagree (3)’ and ‘definitely disagree (4)’. Items are summed up to calculate subscale scores and a total composite score, with higher scores indicating more endorsement of autistic traits.

#### BRIEF-A

The BRIEF-A measures respondents’ own perception of executive functioning in their daily life [34], measuring two domains: *metacognition* and *behavioral regulation*. The questionnaire is composed of 75 items on which respondents indicate how often a described problem has occurred over the past month, with response options ‘never (1)’, ‘sometimes (2)’, or ‘often (3)’. Subscale scores and a total composite score are calculated by summing the scores of the relevant items. A higher score represents poorer executive functions in everyday life. In addition, the BRIEF-A contains three scales to check the validity of answers: *inconsistency*, *negativity*, and *infrequency*. Respondents who scored above the cut-offs defined in the manual on any of the scales were excluded from the sample (*negativity* >3, *inconsistency* >7, *infrequency* >2).

#### DASS-21

The DASS-21 is a questionnaire that measures emotional distress in the domains of *depression*, *anxiety* and *stress* [33]. Each of the three scales contains 7 items, scored on a 4-point Likert scale with response options ‘never (1)’, ‘sometimes (2)’, ‘often (3)’, or ‘very often (4)’. Subscale scores were formed by summing the scores of the relevant items. Higher scores indicate more severe symptoms of emotional distress.

### Data analysis

All analyses were performed using R (version 3.0.2) [36]. Data was checked for normality and linearity; if the assumptions were violated, non-parametric tests were performed. A p-value of 0.05 or less was considered statistically significant.

#### Participant descriptives

The participant characteristics were described with mean and standard deviation (SD). Subsamples were compared using a student’s t-test; proportions were compared using a chi-square test.

#### Confirmatory factor analysis

The items of the SVCq-abi were categorized in alignment with the 5-factor structure of the original SVCq. This created the following structure of the SVCq-abi: *function related* (item 2, 3, 5, 10 and 20), *luminance related* (item 6, 11, 13 and 14), *task related* (item 8, 9, 15, 16 and 22), *altered visual perception* (item 4, 7, 12, 19, 21), and *ocular discomfort* (item 17 and 18).

**Fig 1:**
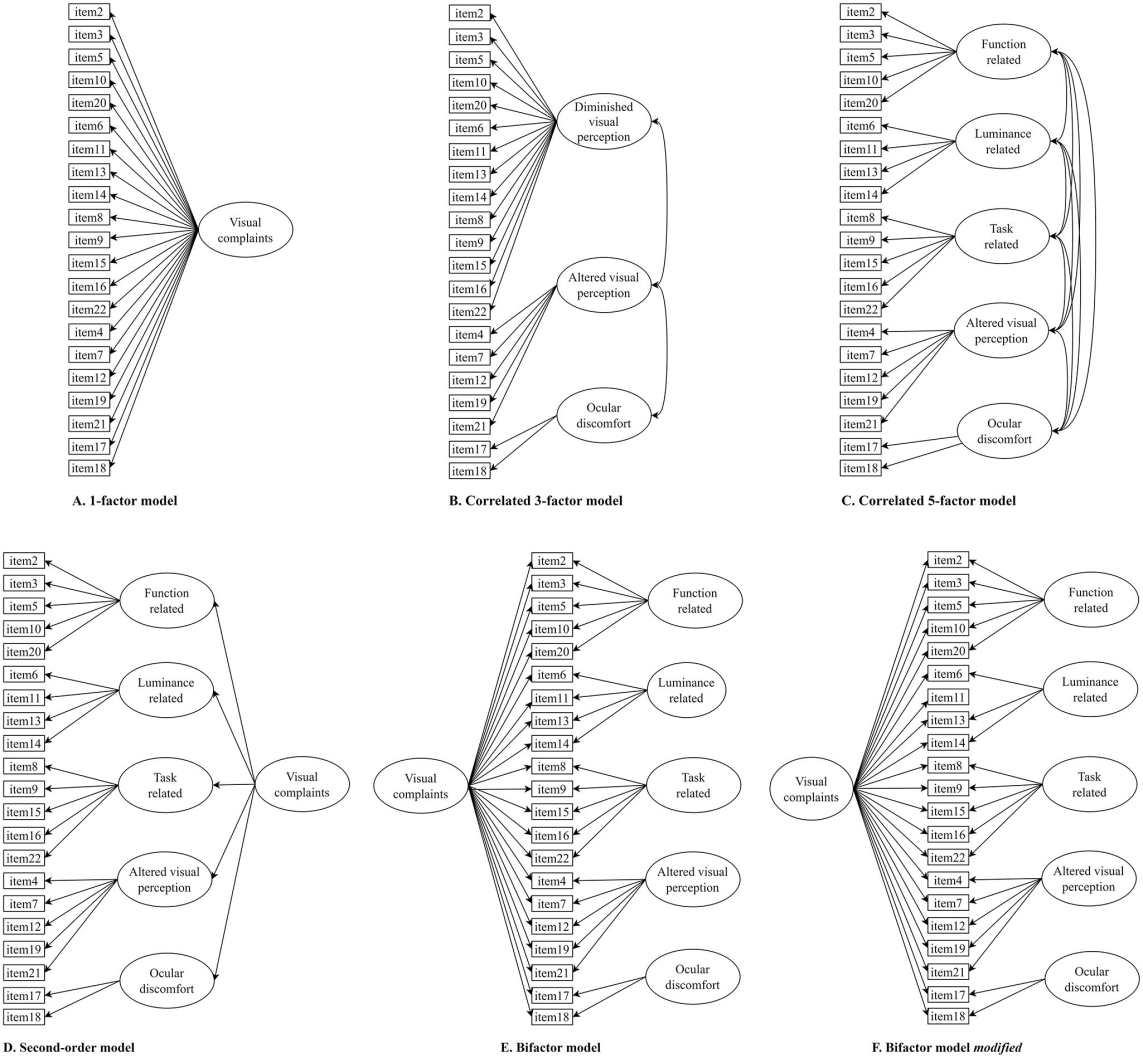
Confirmatory factor analysis models of the SVCq-abi. Models as follows: (A) 1-factor model; (B) correlated 3-factor model; (C) correlated 5-factor model; (D) second-order model; (E) bifactor model; (F) bifactor model *modified*. SVCq-abi = Screening Visual Complaints questionnaire-acquired brain injury.

For factor analysis, the sample was randomly split in two equally sized subsamples. A confirmatory factor analysis (CFA) was performed on subsample 1 to evaluate the fit of the proposed correlated 5-factor structure of the SVCq-abi (see Fig 1C for an illustration). A robust diagonally weighted least squares estimation with mean and variance adjusted test statistic and standard errors was employed (i.e. WLSMV) [37], as implemented in the R package *lavaan* [38]. WLSMV is the suggested estimation method for ordinal non-normal data [39–42]. To optimize model identification variances of the factors were fixed to 1. Additionally, the fit of competing factor models were explored, including a correlated 3-factor model (as proposed by Huizinga et al. [26]; see Fig 1B). Furthermore, we examined three models to test the possibility of a general ‘visual complaints’ factor, including a 1-factor model (see Fig 1A), a second-order model (5 first-order factors, and 1 general second-order factor; see Fig 1D), and a bifactor model (5 specific factors and 1 general factor; see Fig 1E).

Goodness of fit indices were used to ascertain model fit: normed chi-square index (χ^2^ / df), Root-Mean-Square Error of Approximation (RMSEA), Standardized Root Mean-Square Residual (SRMR) and Comparative Fit Index (CFI). We used the normed χ^2^ as this parameter takes sample size into account [43]. A good model fit is indicated when normed χ^2^ values are ≤ 3.0, RMSEA ≤ 0.07 (with the upper limit of the confidence interval ≤ 0.08), SRMR ≤ .08, and CFI ≥ .95 (acceptable if CFI ≥ 0.90) [43–45]. Additionally, item loadings were evaluated, with values above 0.50 deemed adequate for capturing their respective constructs. For nested models, scaled chi-squared difference tests were performed to test for significant differences between the models (significant Δχ^2^) [46]. Non-nested models were compared by evaluating fit statistics. To ensure robustness of the results, CFA of the respective models were repeated in subsample 2 for cross-validation.

#### Item and scale evaluation

We investigated the presence of floor and ceiling effects (< 70% of the respondents endorsing the lowest or highest response category was considered acceptable). Scale reliability was assessed by item-rest correlations (acceptable if > 0.30), inter-item correlations to identify possible item redundancy (acceptable if < 0.80), and internal consistency. Moreover, Average Variance Extracted (AVE) was calculated to measure the proportion of variance explained by each factor, with values above 0.50 indicating good convergence. To evaluate internal consistency, categorical omega (⍵) was calculated in case a first-order structure was selected, defining the amount of variance accounted for by the items that underlie the factor [47–50]. When a second-order or bifactor structure was established, omega hierarchical (⍵_h_) was used [51], which gauges the reliability of the general factor defined in the model adjusting for the specific factors and vice versa. Spearman-Brown’s coefficient was calculated when the factor comprised a 2-item scale [52]. Values of .70 and higher were considered as good internal consistency [53]. Test-retest reliability was evaluated by calculating intraclass correlation coefficients (ICC) between the two measurements, using a two-way mixed-effects model with absolute agreement. ICC values between 0.5 and 0.75 indicate moderate reliability, values higher than 0.75 indicate good reliability [54].

#### Convergent and divergent validity

To establish convergent validity, the correlation between the SVCq-abi and the CVSQ was determined. As both questionnaires measure a similar construct, we expected moderate to large correlations. To evaluate divergent validity, we correlated SVCq-abi scores with the AQ-short, DASS-21 and BRIEF-A. Divergent validity was assessed under the assumption that these questionnaires measure a different construct than the SVCq-abi. We expected small correlations with the DASS-21 (visual complaints vs mental health) and the BRIEF-A (visual complaints vs executive dysfunction). For the AQ-short, which was assumed to measure an even more distinct construct (visual complaints vs autistic traits), we expected negligible correlations. All associations were established by Spearman’s correlation coefficients, and evaluated following Cohen’s criteria (r < 0.1, negligible; 0.1 ≤ *r* < 0.3, small; 0.3 ≤ *r* < 0.5, moderate; *r* ≥ 0.5, large; [55]). To adjust for multiple hypotheses testing, we used false discovery rate (FDR) adjusted p-values [56].

## Results

### Participant descriptives

Table 1 shows the characteristics of the study population. In total, 1159 participants were considered for inclusion. The two measurements of one participant were excluded due to double responses (n = 2). In addition, 142 participants were excluded based on the validity scales of the BRIEF-A, due to negative tendency (n = 30), inconsistency (n = 11), and infrequency (n = 100) of responses. After exclusion, 1021 were left for data analysis. For factor analyses, no significant differences were found between subsample 1 and subsample 2 with regard to age, gender, education level, presence of ophthalmic condition, and presence of neurological condition (Table 1).

**Table 1:** Characteristics of the study population.

	Total sample	Subsample 1	Subsample 2	p-value
N	1021	511	510	
Gender—female, n (%)	446 (44%)	232 (45%)	214 (42%)	0.31
Age (years)				0.84
Mean [SD]	60.2 [15.5]	60.3 [15.3]	60.1 [15.7]	
Range	18–94	20–93	18–94	
Education^a^, n (%)				0.87
Low	132 (13%)	67 (13%)	65 (13%)	
Medium	520 (51%)	256 (50%)	264 (52%)	
High	369 (36%)	188 (37%)	181 (35%)	
Presence of severe ophthalmic condition^b^, n (%)	36 (4%)	19 (4%)	17 (3%)	0.87
Presence of neurological condition, n (%)	80 (8%)	40 (8%)	40 (8%)	1.00

SD = standard deviation.

^a^ = based on the International 2011 Standard Classification of Education [57].

^b^ = including glaucoma, age-related macular degeneration, diabetic retinopathy, and ocular trauma.

^c^ = including stroke, infection, trauma, tumor, anoxia, dementia, multiple sclerosis, and Parkinson’s disease.

### Self-reported visual complaints

On the first item of SVCq-abi, 587 (57%) of the 1021 participants reported at least one visual complaint. In total, 644 visual complaints were reported, of which 522 (81%) were similar to the 21 complaints stated in the SVCq-abi. The most frequently reported visual complaints not covered by the SVCq-abi included: spots or floaters in the eye (n = 36), tearing of the eyes (n = 27), and difficulty watching a digital screen (n = 15).

### Factor analyses

Table 2 presents the goodness of fit statistics of the CFA models of both subsamples. In subsample 1, the 1-factor, correlated 3-factor, correlated 5-factor and second-order model all demonstrated good fit statistics. The initial bifactor model did not produce an admissible solution and showed problematic negative standardized item loadings. Notably, item 11 showed an excessively high loading (λ = 228.4) in factor 2 and negative residual variance (σ^2^ = -52169.4). Fit statistics of the initial model are therefore not shown in Table 2. Modifications were made by constraining all item loadings above zero and excluding item 11 from factor 2 (see Fig 1F for an illustration), resulting in an admissible bifactor model with good model fit (Table 2). While some item loadings in the modified bifactor model did not meet significance level, this was likely due to underpowered analyses, as this issue was resolved when repeating CFA of the modified bifactor model in the complete sample. Cross-validation in subsample 2 confirmed good fit for all proposed models, including the modified bifactor model (Table 2).

**Table 2:** Fit indices of the confirmatory factor analyses of the SVCq-abi in subsample 1 and subsample 2.

	χ^2^ (df)	χ^2^/df	RMSEA [95% CI]	SRMR	CFI
**Subsample 1 (n = 511)**					
1-factor model	573.262 (189)	3.03	0.063 [0.057–0.069]	0.080	0.915
Correlated 3-factor model	518.914 (186)	2.79	0.059 [0.053–0.065]	0.076	0.926
Correlated 5-factor model	444.436 (179)	2.48	0.054 [0.048–0.060]	0.069	0.941
Second-order model	458.723 (184)	2.49	0.054 [0.048–0.060]	0.071	0.939
Bifactor model *modified*	359.870 (169)	2.13	0.047 [0.040–0.054]	0.063	0.958
**Subsample 2 (n = 510)**					
1-factor model	631.909 (189)	3.43	0.068 [0.062–0.074]	0.076	0.936
Correlated 3-factor model	573.176 (186)	3.08	0.064 [0.058–0.070]	0.072	0.944
Correlated 5-factor model	458.464 (179)	2.56	0.055 [0.049–0.062]	0.064	0.960
Second-order model	493.786 (184)	2.68	0.058 [0.051–0.064]	0.068	0.955
Bifactor model *modified*	420.231 (169)	2.49	0.054 [0.048–0.061]	0.062	0.964

SVCq-abi = Screening Visual Complaints questionnaire-acquired brain injury; df = degrees of freedom; RMSEA = Root Mean Squared Error of Approximation; CI = confidence interval; SRMR = Standardized Root Mean Square Residual; CFI = Comparative Fit Index.

#### Model comparison

In subsample 1, the 5-factor model showed a good fit, and outperformed the 3-factor model (Δχ^2^ (7) = 67.5, p < .001). Covariances above 0.7 were found amongst the five factors, indicating potential evidence for a general (higher-order) factor that describes the correlations. Although the second-order model showed a good fit, a significant decrease in fit was found compared to the 5-factor structure (Δχ^2^ (5) = 19.8, p = 0.001). Moreover, fit of the 1-factor model was significantly worse than the 5-factor model (Δχ^2^ (10) = 111.1, p < .001). Similar results were found in subsample 2, with the correlated 5-factor model outperforming the 1-factor model (Δχ^2^ (10) = 151.4, p < .001), 3-factor model (Δχ^2^ (7) = 98.6, p < .001), and second-order model (Δχ^2^ (5) = 34.3, p < 0.001).

The 5-factor model was not nested within the modified bifactor model, which prohibited χ^2^ comparisons. The modified bifactor model exhibited superior fit across all indices in both subsamples.

#### Model selection

Although the bifactor model showed superior fit, the fit indices of the correlated 5-factor model were comparable with only approximately 1% difference in CFI. This marginal difference questioned if the improved model fit outweighs the drawbacks associated with the bifactor model. Most importantly, to attain an admissible solution of the bifactor model we had to constrain the item loadings and remove item 11 from factor 2. However, given that item 11 theoretically aligns with factor 2, and showed no issues in other CFA models, this was not deemed preferable. Additionally, we favored a lenient item deletion stance since our sample represents a community sample instead of the target population. Lastly, a superior fit of the bifactor model could be expected considering it estimates more parameters [58,59]. Consequently, we favored a 5-factor structure over a bifactor structure for the SVCq-abi. Item loadings of the final 5-factor structure for the total sample are displayed in Table 3. Subscale scores of the 5 factors were calculated by summing up the relevant items.

**Table 3:** Response distribution, item loadings of the 5-factor model, item-rest correlation, internal consistency, AVE and test-retest reliability of the SVCq-abi.

Scale and item content	Response distribution over response categories (%)			Item loadings	Item-rest correlation	Internal consistency	AVE	Test-retest reliability ICC [95% CI]
	1	2	3					
*Function related*						0.78*	0.51	0.73 [0.65–0.80]
2: Unclear vision	28%	54%	19%	0.69	0.57			
3: Reading	43%	38%	18%	0.62	0.50			
5: Trouble focusing	45%	44%	11%	0.77	0.57			
10: Contrast sensitivity	55%	35%	10%	0.73	0.51			
20: Depth perception	72%	22%	6%	0.76	0.38			
*Luminance related*						0.75*	0.58	0.65 [0.55–0.74]
6: Blinded by light	52%	35%	13%	0.70	0.47			
11: Light adjustment	63%	30%	7%	0.79	0.60			
13: Needing more light	56%	30%	14%	0.68	0.48			
14: Dark haze	82%	15%	3%	0.85	0.49			
*Task related*						0.80*	0.66	0.72 [0.63–0.79]
8: Traffic	72%	23%	5%	0.73	0.55			
9: Looking for something	82%	15%	3%	0.83	0.62			
15: Mobility	88%	11%	2%	0.83	0.60			
16: Needing more time	70%	25%	5%	0.82	0.59			
22: Grabbing something	90%	9%	1%	0.83	0.50			
*Altered visual perception*						0.75*	0.58	0.69 [0.59–0.77]
4: Double vision	82%	15%	3%	0.74	0.49			
7: Color vision	88%	9%	3%	0.74	0.44			
12: Distorted images	88%	9%	3%	0.86	0.58			
19: Visual hallucinations	71%	24%	5%	0.68	0.47			
21: Visual field	85%	11%	4%	0.78	0.52			
*Ocular discomfort*						0.62^†^	0.65	0.71 [0.61–0.78]
17: Painful eyes	77%	19%	3%	0.91	0.35			
18: Dry eyes	58%	29%	13%	0.69	0.35			

SVCq-abi = Screening Visual Complaints questionnaire-acquired brain injury; AVE = Average Variance Extracted; ICC = Intraclass Correlation Coefficients.

* = established with categorical omega.

^†^ = established with Spearman Brown’s coefficient.

### Item and scale evaluation

Employing the 5-factor model, floor effects were found for item 20, item 14, all items in the *task related* subscale (item 6, 11, 13 and 14), the *altered visual perception* subscale (item 4, 7, 12, 19, and 21), and for item 17, as shown in Table 3. No ceiling effects were found. All item-rest correlations exceeded 0.3 and inter-item correlations did not surpass 0.80 (range 0.20–0.51) within the subscales. AVE values were above 0.5 for all subscales. Furthermore, all subscales showed good internal consistency, except for *ocular discomfort*, which showed insufficient internal consistency (Table 3).

With regard to test-retest reliability, of the 157 participants that completed the reassessment of the SVCq-abi, 14 were excluded based on BRIEF-A validity scales, due to a negativity tendency (n = 1), inconsistency (n = 1), or infrequency (n = 12) of responses. The retest was completed after 19 ± 11 days (range 12–28, median 20 days). As illustrated in Table 3, good test-retest reliability was found for the *function related*, *task related* and *ocular discomfort* subscales. *Luminance related* and *altered visual perception* showed moderate test-retest reliability.

### Convergent and divergent validity

Table 4 presents the correlation coefficients between the SVCq-abi and the convergent and divergent measures. Regarding convergent validity, all correlations with the CVSQ were significant after FDR multiple hypothesis testing adjustment (*p*_adj_ < 0.001) and ranged from moderate to strong. For divergent validity, the SVCq-abi showed significant correlations with the BRIEF-A and DASS-21, with effect sizes ranging from weak to moderate. Between the SVCq-abi and the AQ-short roughly half of the correlation were insignificant and all effect sizes were considered negligible.

**Table 4:** Spearman’s correlation coefficients between the SVCq-abi, the CVSQ, the BRIEF-A, the DASS-21 and the AQ-short.

	SVCq-abi				
	*Function related*	*Luminance related*	*Task related*	*Altered visual perception*	*Ocular discomfort*
Convergent measures					
CVSQ *Kerkhoff-part*	0.57*	0.55*	0.45*	0.43*	0.32*
CVSQ *Dittrich-part*	0.36*	0.35*	0.47*	0.38*	0.30*
CVSQ *total*	0.50*	0.50*	0.54*	0.48*	0.37*
Divergent measures					
BRIEF-A *behavioral regulation*	0.30*	0.27*	0.34*	0.30*	0.21*
BRIEF-A *metacognition*	0.26*	0.24*	0.29*	0.24*	0.17*
BRIEF-A *composite*	0.30*	0.27*	0.33*	0.29*	0.21*
DASS-21 *stress*	0.31*	0.32*	0.36*	0.31*	0.31*
DASS-21 *anxiety*	0.32*	0.35*	0.36*	0.36*	0.35*
DASS-21 *depression*	0.29*	0.29*	0.33*	0.28*	0.25*
AQ-short *numbers/patterns*	-0.03	-0.08*	-0.08*	-0.12*	-0.07*
AQ-short *social behavior*	-0.04	-0.05	-0.04	-0.05	-0.06
AQ-short *total*	-0.04	-0.07*	-0.06	-0.08*	-0.08*

SVCq-abi = Screening Visual Complaints questionnaire-acquired brain injury; CVSQ = Cerebral Visual Symptoms Questionnaire; BRIEF-A = Behavior Rating Inventory of Executive Function-Adult; DASS-21 = Depression Anxiety Stress Scale-21; AQ-short = Autism-spectrum Quotient-short.

* = significant after FDR multiple hypothesis testing adjustment.

## Discussion

The present study developed and evaluated the psychometric properties of the SVCq-abi, a screening tool for assessing visual complaints in individuals with ABI. The SVCq-abi is an adaptation of the SVCq; a questionnaire initially designed for individuals with neurodegenerative diseases. Following CFA, a correlated 5-factor model was favored over a 1-factor, correlated 3-factor, second-order, and bifactor model. Floor effects were found in various items of the SVCq-abi. Furthermore, through the evaluation of item-rest correlations, inter-item correlations, AVE and internal consistency, the subscales showed moderate to good scale reliability. With the exception of *ocular discomfort*, which showed insufficient internal consistency. We found moderate to good test-retest reliability of the subscales. Furthermore, moderate to large correlations were established with the convergent measures, and negligible to moderate correlations with the divergent measures.

We confirmed a 5-factor structure of the SVCq-abi in our community sample, aligning with previous findings on the structure of the SVCq [27,28]. In contrast, we employed WLSMV instead of DWLS as an estimation method, as WLSMV has been advised over DWLS when 3 response categories are used [39]. Furthermore, in our study, we evaluated competing models, including the bifactor model recommended by Chen and Zhang [60]. Although the bifactor model showed superior fit, the difference in fit indices with the 5-factor model was marginal, with approximately 1% difference in CFI. Therefore, we favored a 5-factor structure over a bifactor structure for the SVCq-abi due to several practical concerns associated with the bifactor model. Of particular significance was the problematic removal of item 11 from factor 2. This was not deemed preferable due to its theoretical alignment, and the fact it showed no issues in other CFA models. In addition, we took a cautious approach to item deletion considering it is a community sample.

No ceiling effects were found. However, approximately half of the items exhibited floor effects, indicating reduced sensitivity of these items in capturing variability among individuals with fewer visual complaints. Given our aim is to screen for individuals who experience more visual complaints, these floor effects could be less concerning in a community sample. Overall, the SVCq-abi demonstrated moderate to good scale reliability and test-retest reliability, similar to the original SVCq [26]. Small differences between the studies could be attributed to item adaptations and population variation. Notably, the *ocular discomfort* subscale of the SVCq-abi demonstrated insufficient internal consistency, potentially due to its two-item nature which may pose a threat for scale reliability [52,61]. Furthermore, test-retest reliability was moderate for *luminance related* and *altered visual perception*. Moderate reliability might pose issues for use in clinical practice, where repeatability of scores is essential.

In support of convergent validity, a higher level of visual complaints reported on the SVCq-abi corresponded with a higher level of visual complaints reported on the CVSQ, demonstrated by moderate to strong correlations. Notably, the CVSQ measures responses on a binary scale, which may influence the correlation strength with the SVCq-abi comprising 3 response options. Lowest correlations were observed between our questionnaire and the Dittrich-part of the CVSQ. This is potentially due to the fact that the Dittrich-part measures activity-related problems without specifically relating it to vision. Furthermore, the SVCq-abi subscale *ocular discomfort* showed relatively low correlations with the CVSQ. Such results were foreseeable as these items focus on eye comfort and health, and do not capture visual complaints on a functional level like the CVSQ. Huizinga et al. [26] evaluated convergent validity of *ocular discomfort* by its correlation with the *ocular pain* subscale of National Eye Institute Visual Functioning Questionnaire-25 [62] and found a strong association.

Divergent validity was assessed on the assumption that the SVCq-abi and the divergent questionnaires measure different psychological constructs. We observed negligible to moderate associations with the divergent measures. As hypothesized, particularly low associations were found with autistic traits reported on the AQ-short. Roughly half of the correlations of the SVCq-abi with the BRIEF-A and the DASS-21 were of moderate strength. In particular, the *task related* subscale showed mostly moderate correlations. The known association between executive dysfunction and emotional distress with visual functioning and performing tasks may have contributed to the strength of these correlations [63–70]. Additionally, an explanation may be the phenomenon of a general pathology factor that explains the endorsement of complaints across all measured domains [71]. The original SVCq showed similar correlations with the BRIEF-A and the DASS-21 [26]. Yet, associations in our sample appeared slightly higher, possibly due to the older age of our participants [72–75]. Nonetheless, we expected the association with the convergent measures to be higher than the divergent measures. In total, the SVCq-abi shared 9% to 32% of variance with the convergent measures, and 2% to 13% with the divergent measures. Taken together, evidence for convergent and divergent validity was considered minimal, but sufficient.

This study has some strengths and limitations. A strength is the exploration of competing factor models, adding to the robustness of the initial 5-factor structure. However, the factor structure, as well as the psychometric properties, were established in a community sample. It is unknown to which extent these properties can be generalized to individuals with ABI. Notably, the prevalent floor effects in half of the items may have distorted correlational structures, which potentially limits the applicability of the findings to a clinical sample. Therefore, further exploration of the psychometric properties in a sample with ABI is an important issue for future studies. Another limitation is that we employed a Dutch adapted version of the CVSQ as a convergent measure. Although the original CVSQ has been extensively validated, the Dittrich-part and the Dutch translation has not been validated yet, which may have influenced convergent validity results. Lastly, we used the scale-shifted fit indices of the WLSMV estimation method for CFA to enhance comparability with prior research, given it is the default method in most factor analysis software. However, although the RMSEA and CFI indices are widely applied with WLSMV, recent developments have raised concerns that the model fit is overestimated with these indices [76,77]. These findings raise questions about the robustness of our CFA results and it is essential to acknowledge that the field is evolving.

The SVCq-abi may be of benefit to individuals with ABI, and professionals within rehabilitative practice. It could offer a valuable and pragmatic method to screen for visual complaints, providing insights into various aspects of visual impairment. Unlike objective vision measurements and screening tests, the SVCq-abi requires minimal time and is easy to administer. Based on the current findings, we suggest making use of the 5-factor model for the SVCq-abi. In addition to the evaluation of the subscale scores, an evaluation of the individual items is highly recommended for additional insights into specific complaints and needs. Additionally, item 23 may offer insight into the extent of limitations experienced, and help guide further care. It is important to note that some validity and reliability issues of the SVCq-abi remain. These concerns require further exploration in an ABI sample to ensure the robustness and utility of the SVCq-abi in a clinical setting. In conclusion, the SVCq-abi shows fundamental psychometric properties and a robust 5-factor structure. It appears a valid tool to measure visual complaints in a community sample, which holds promise for use in individuals with ABI. Some validity and reliability issues emerged which could pose issues for use in clinical practice. Hence, further validation in individuals with ABI is essential.

## Supporting information

S1 Appendix The Screening Visual Complaints questionnaire-acquired brain injury [original Dutch questionnaire]. (PDF)

S2 Appendix The Screening Visual Complaints questionnaire-acquired brain injury [English translation]. (PDF)
